# Inebriety: Its Etiology, Pathology, Treatment and Jurisprudence

**Published:** 1889-10

**Authors:** 


**Inebriety: Its Etiology, Pathology, Treatment and Jurisprudence.** By NORMAN KERR, M. D., F. R. S.; Fellow of the Medical Society of London; President Society for the Study of Inebriety; Chairman British Medical Association Inebriates' Legislation Committee; Consulting Physician Dalrymple Home for the Treatment of Inebriety; Corresponding Secretary American Association for the Cure of Inebriates. Second edition. Cloth; 12mo. London: H. K. Lewis, 136 Gower street, W. C. 1889. Pp. xxxii.—471.

This is the most complete monograph which has appeared on this important subject. Inebriety is here discussed in all its forms, complications, and sequelæ. The treatment of the habit of the drunken conditions of mania-a-potu, delirium-tremens, of the craving for liquor, is given with great clearness, and with the positiveness that comes only from experience.

The chapters devoted to the medico-legal aspects of inebriety are interesting, and an able exposition of the views of such men as Dr. Crothers and others, who wish to make inebriety a legal excuse for crime.

The marginal notes on every page, and the very complete index, make the finding of any special subject an easy matter. It is well set up, and is free from typographical errors. J. W. P.

